# Individualized identification of sexual dysfunction of psychiatric patients with machine-learning

**DOI:** 10.1038/s41598-022-13642-y

**Published:** 2022-06-10

**Authors:** Yang S. Liu, Jeffrey R. Hankey, Stefani Chokka, Pratap R. Chokka, Bo Cao

**Affiliations:** 1 Chokka Center for Integrative Health, 301 - 2603 Hewes Way NW, Edmonton, AB T6L 6W6 Canada; 2 Department of Psychiatry, Faculty of Medicine & Dentistry, University of Alberta, Edmonton, AB T6G 2B7 Canada; 3 Department of Psychology, York University, Toronto, Canada

**Keywords:** Psychiatric disorders, Depression, Risk factors, Machine learning

## Abstract

Sexual dysfunction (SD) is prevalent in patients with mental health disorders and can significantly impair their quality of life. Early recognition of SD in a clinical setting may help patients and clinicians to optimize treatment options of SD and/or other primary diagnoses taking SD risk into account and may facilitate treatment compliance. SD identification is often overlooked in clinical practice; we seek to explore whether patients with a high risk of SD can be identified at the individual level by assessing known risk factors via a machine learning (ML) model. We assessed 135 subjects referred to a tertiary mental health clinic in a Western Canadian city using health records data, including age, sex, physician’s diagnoses, drug treatment, and the Arizona Sexual Experiences Scale (ASEX). A ML model was fitted to the data, with SD status derived from the ASEX as target outcomes and all other variables as predicting variables. Our ML model was able to identify individual SD cases—achieving a balanced accuracy of 0.736, with a sensitivity of 0.750 and a specificity of 0.721—and identified major depressive disorder and female sex as risk factors, and attention deficit hyperactivity disorder as a potential protective factor. This study highlights the utility of SD screening in a psychiatric clinical setting, demonstrating a proof-of-concept ML approach for SD screening in psychiatric patients, which has marked potential to improve their quality of life.

## Introduction

Sexual dysfunction (SD) is a persistent, distressing change in desire, arousal, orgasmic function, or pain during intercourse^1–5^. Negative impacts of SD may include stress in romantic relationships, disruptions to work, and job loss^6^. Risk factors for both men and women’s SD are multifaceted, including physical illnesses such as diabetes, heart diseases, urinary tract disorders, other chronic illnesses and socio-cultural factors^7,8^, as well as substance abuse, psychiatric disorders^7,8^ and unhealthy life-style^9^. SD is very common among patients presenting with mental health issues^1–5,10^ and especially prevalent among those with major depressive disorder (MDD)^11–14^, which is projected to be the most burdensome disease worldwide by 2030^15^. Despite its prevalence, SD in psychiatric patients is under-diagnosed and frequently missed in primary care^4,6,14^. It is particularly challenging to differentiate SD among patients with affective disorders as a pre-existing condition from those with treatment-emergent sexual dysfunction (TESD)^16^, a common side effect of antidepressant medication such as Selective serotonin reuptake inhibitors (SSRIs) and Serotonin and norepinephrine reuptake inhibitors (SNRIs). However, no matter whether SD is associated with primary conditions or the treatment thereof, SD often leads to treatment noncompliance or discontinuation^12,17^. In one patient survey, SD was reported as a primary culprit for antidepressant treatment noncompliance and was considered “extremely difficult to live with”^9^. Thus, recognition of SD at various points of contact with the healthcare system may enable the selection of more appropriate treatment options for SD and/or other primary diagnoses with favorable side effect profiles and promote treatment compliance^9,13^.

In addition to well-known etiological factors associated with high rates of SD, such as MDD and taking prescription medication that may induce TESD, sex is a common demographical factor that may inform the risk of SD, which disproportionally affects women compared to men. For example, a study from 1992 revealed that 43% of women reported SD as opposed to 31% of men^2^, and this pattern has been supported by more recent literature^1,14,18,19^. Psychiatric diagnoses, recent drug treatment, and demographics such as sex and age are all routinely accessible at health care facilities as a part of Electronic Health Records (EMR). Therefore, computation of widely available risk factors may be a practical way to enable the proactive identification of SD.

Modeling based on machine learning (ML) is gaining traction in psychiatry^20^ and has been successfully applied in mood disorder screening^21–24^. ML-based methods are also widely explored in the broader contexts of personalized medicine and healthcare utilizing EMR data^25,26^. ML can be viewed as an extension of the traditional statistics approach, with data-driven algorithms optimized for predictive accuracy^27^. Benefits of ML include compatibility with large, multidimensional, correlated data, identifying important features driving predictive performance, reducing overfitting, and improving model generalizability using validation techniques^27^. ML-based models are compatible with input from big data such as EMR records, and can make better individual patient-level predictions of disease risks compared to traditional statistical methods^28^. Accordingly, ML methods are optimal for achieving our goal of facilitating clinically relevant, accurate and scalable SD screening.

In this cross-sectional, naturalistic study, we assessed the prevalence of SD in a tertiary psychiatric care setting and explored whether individual patients with a high risk of SD, assessed by a positive screening on the Arizona Sexual Experience Scale (ASEX)^29–31^, could be identified proactively using known risk factors such as sex, MDD and drug treatment using a ML model. We further explored other understudied but common psychiatric diagnoses and drug treatments that may contribute to SD.

## Methods

### Patients

The study was approved by the University of Alberta Health Research Ethics Board (Pro00072946), and all research was performed in accordance with the Declaration of Helsinki. Over a 22-week period, 135 subjects were referred to a mental health clinic. These subjects, all of whom provided written consent, were eighteen years or older and evaluated with the ASEX^29–31^. Data from two participants were excluded from analysis due to missing ASEX scores. For the 133 eligible subjects (Mean Age = 39.4; 51 males and 82 females), 58 patients (43.6%; 18 males and 40 females) met the diagnostic criteria for MDD, while 72 patients (54.1%; 19 males and 53 females) met the criteria for SD (see Table S1 for a full list of patient characteristics of the SD and non-SD groups).

### Study design and assessments

This was a single-centre, naturalistic study conducted at a tertiary clinic in a Western Canadian city. Self-rated intake assessment data included age, sex, and completion of the ASEX. Our data also included diagnoses of MDD, bipolar disorder (BD), generalized anxiety disorder (GAD), attention deficit hyperactivity disorder (ADHD), and borderline personality disorder (BPD) based on the DSM-IV-TR and confirmed by a board-certified psychiatrist.

The ASEX has well-established internal consistency, test–retest reliability, and convergent and discriminant validity for assessing sexual functioning in a clinical setting^29–31^. It is also concise and user-friendly, consisting of five items, with a 6-point Likert scale comprising each. Possible total scores range from 5 to 30, with higher scores indicating more sexual dysfunction. We used the recommended cut-off methods, with a total ASEX score of > 19, any one item with a score of > 5, or any three items with a score of > 4 indicating sexual dysfunction^29^.

### Data analysis

We adopted a ML approach to data analysis focusing on the classification of SD and identifying predictive risk factors, also referred to as predictive features, of SD. Machine learning analysis was conducted using Python 3.6 with Scikit-Learn 0.22.1. The outcome variable in each case was the presence or absence of SD. Except for age, coded as a continuous variable, all candidate features for making predictions were binary coded (1 = Yes, 0 = No) for female sex, MDD, ADHD, BD, BPD, as well as taking SSRIs, non-psychiatric medication, antidepressants other than SSRIs, stimulants, benzodiazepines or hypnotics, antipsychotics or anticonvulsants, or no medication.

ML algorithms such as logistic ridge regression with leave-one-out cross-validation (LOOCV) and cross-validation-based hyperparameter tuning have the advantage of being data-driven and results-focused, reducing over-fitting and model biases^32–34^. The LOOCV procedure takes n iterations; for each iteration one sample is reserved for model testing and n-1 samples for model training, so the procedure always evaluates the learned model’s performance on unknown data and LOOCV performance such as accuracy, the area under the receiver operating characteristic curve (area under the ROC curve or AUC), sensitivity and specificity, reflecting a collection of the individual model’s prediction performances on validation data. Hyperparameter tuning using k-fold cross-validation, at each iteration of LOOCV model training, is also referred as internal cross-validation. The use of multiple layers of cross-validation, e.g., an external LOOCV cross-validation in conjunction with an internal five-fold cross-validation for hyperparameter tuning, is referred as nested cross-validation, and has been recommended for small sample pilot studies for unbiased estimates^34,35^. Thus, this combined approach enables us to make confident classifications at an individual patient level.

For all analyses, features were used to predict SD using logistic ridge regression. We used a LOOCV procedure for external cross-validation, where each subject was reserved for testing once, while the logistic ridge model was trained on the remaining 132 subjects. For each LOOCV iteration, hyperparameter turning was performed based on tenfold internal cross-validation, optimizing for balanced accuracy, representing the average value of sensitivity and specificity. To provide a baseline comparison, we also fitted logistic regression instead of logistic ridge regression, using an identical LOOCV procedure.

The importance of features was evaluated by ranking the average coefficient values across 133 LOOCV iterations. The coefficient values cannot be interpreted beyond relative importance due to the regularization, thus we conducted post hoc Pearson’s correlation analysis between each feature and SD to supplement the model’s interpretation.

### Informed consent

Informed consent was obtained from all subjects and/or their legal guardian(s).

## Results

### Classification performance

**Figure 1:**
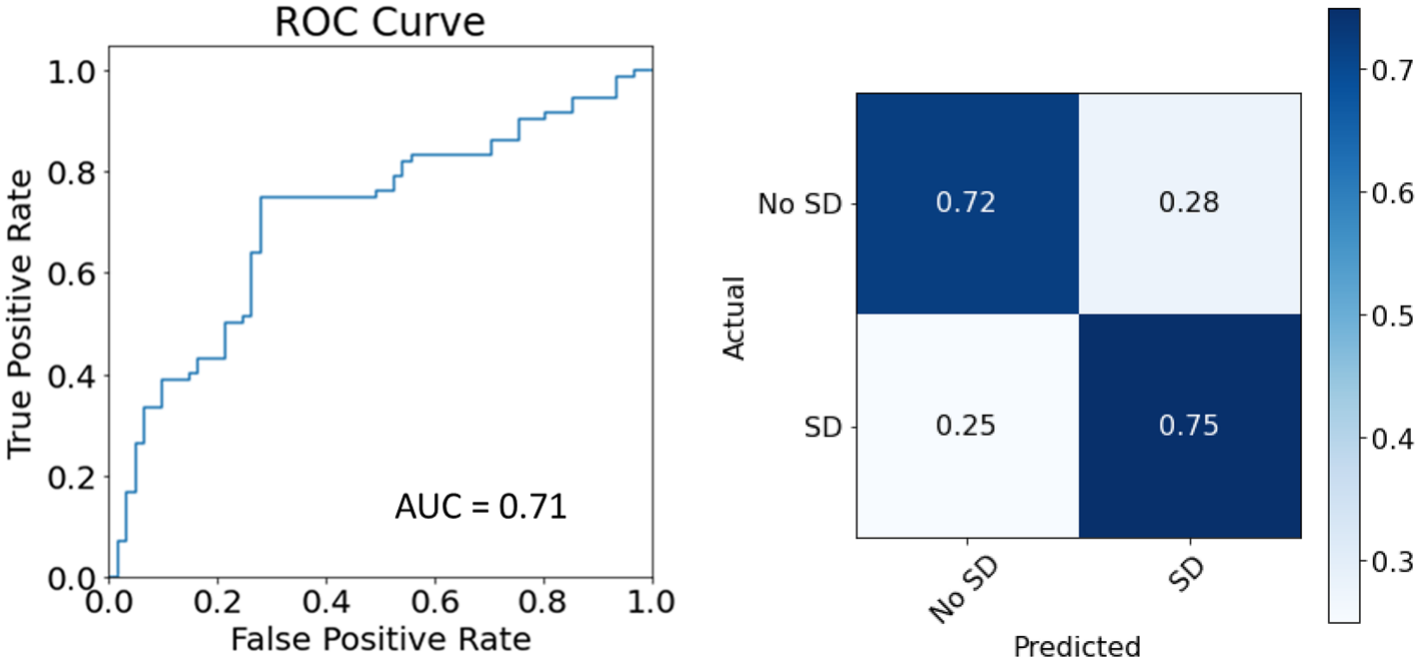
Receiver operating characteristics curve and confusion matrix.

The LOOCV performance of logistic ridge regression model reached a balanced accuracy of 0.736, the average of sensitivity (0.750) and specificity (0.721), outperforming the baseline model following the same LOOCV procedure but with non-regularized logistic regression (i.e., balanced accuracy, sensitivity, specificity = 0.645, 0.667, 0.623, respectively). The positive predictive value and negative predictive value for SD are 0.761 and 0.710, respectively (see Fig. 1). Thus, 75% of SD patients were correctly identified and 76% of patients classified as SD had SD.

Receiver operating characteristics curve and confusion matrix for the classification of SD. True Positive Rate is the rate model classifying a patient with SD as SD, whereas False Positive Rate is the rate model classifying a patient with SD as non-SD. AUC denotes area under the ROC curve.

### Predictive features of SD

The top ranked predictive features for SD, derived from the average absolute values of coefficients from the logistic ridge regression models, were identified as MDD, female sex, and ADHD. In the post hoc correlation analysis, MDD and female sex were positively associated with SD, whereas ADHD was negatively associated with SD (see Table 1 for a summary). Although all other features were not statistically significant in their correlation with SD, the associations were in the expected direction (Table S2). Non-psychiatric medication, benzodiazepines or hypnotics were positively associated with SD whereas no medication and BD were negatively associated with SD. The non-significant features collectively contributed to the predictive performance. A follow-up analysis using an identical LOOCV procedure with a model learned using MDD, female sex, and ADHD as features achieved a balanced accuracy of 0.684, with a sensitivity of 0.778 and specificity of 0.590. Thus, although the three top features can achieve a reasonable performance of SD identification in our sample, other features including age, BD, GAD and BPD diagnoses and drug use information account for a 5% improvement on predictive balanced accuracy. We further confirmed that when all drug-related features were removed from the feature set, LOOCV resulted in a balanced accuracy of 0.696, with a sensitivity of 0.736 and specificity of 0.656; thus, including drug-related features alone contributed to a 4% improvement on predictive balanced accuracy (Table 2).

**Table 1:** SD prevalence for MDD and ADHD.

Sex	Diagnoses	n	with SD (%)
Male n = 51, 37.3% with SD M_age_ (Std) = 39.7 (13.2)	No MDD or ADHD	14	28.6
	ADHD	19	15.8
	MDD	15	80.0
	MDD and ADHD	3	0.0
Female n = 82, 64.6% with SD M_age_ (Std) = 35.8 (11.8)	No MDD or ADHD	23	52.2
	ADHD	19	47.4
	MDD	32	78.1
	MDD and ADHD	8	87.5

Prevalence of SD by Sex, the diagnosis of MDD, ADHD. M denotes mean, SD denotes standard deviation.

**Table 2:** Feature rankings.

Features	Ranking	Averaged coefficient	r^2^	Adjusted *p*
MDD	1	0.45	0.38	< 0.001***
Sex (Female)	2	0.35	0.27	0.013*
ADHD	3	− 0.22	− 0.24	0.030*
Non-psychiatric medication	4	0.10	0.13	0.390
No medication	5	− 0.09	− 0.12	0.420
Benzodiazepines or hypnotics	6	0.08	0.08	0.491
BD	7	− 0.08	− 0.10	0.487
Stimulants	8	0.06	0.00	0.983
GAD	9	0.05	0.07	0.491
BPD	10	0.04	0.07	0.491
Antipsychotics or anticonvulsants	11	− 0.04	− 0.02	0.876
SSRIs	12	0.03	0.09	0.487
Age	13	0.02	0.18	0.129
Other antidepressant	14	− 0.02	0.07	0.491

***p < 0.001, *p < 0.05. *p* values were adjusted using Benjamini & Yekutieli’s method to control for multiple comparison^36^.

## Discussion

Our findings show a proof of concept for SD identification using commonly available patient information, such as a MDD or ADHD diagnosis and sex, with a ML model. In a clinical setting, the classification result could be used to identify patients with potential undiagnosed SD and remind physicians to further validate and incorporate the insights in diagnosis and treatment. The model achieved a balanced accuracy of 0.737 using LOOCV, demonstrating an acceptable performance (AUC between 0.70 and 0.80) within the context of medical diagnostic test assessments^37^. The study shows converging evidence that SD is highly prevalent in mental health patients from a tertiary mental health clinic, especially among MDD patients (78.7% with SD) and among women (64.6% with SD). Patients with ADHD are at lower risk of SD compared to patients without ADHD. In patients with depressive disorders, one study reported a loss of libido or sexual desire in 72% of patients with unipolar depression and 77% of patients with bipolar depression^38^. Further comparative studies confirm a high level of sexual dysfunction in depression patients compared to non-depressed controls^38–40^. Our findings augment the literature investigating the relationship between depression and sexual dysfunction, with further evidence supporting the link between the two disorders in a clinical environment. Furthermore, the findings suggest that females may encounter a higher rate of SD in our outpatient sample referred for treatment for mental health concerns, in line with other SD prevalence studies that have found SD in an estimated 40.9% of women^41^. In addition to identifying MDD and female sex as reliable predictive features of SD, our results demonstrate preliminary evidence that psychotropic medications are also important predictive risk factors of SD in clinical settings. Overall, more than half of our clinical sample, including nearly two-thirds of females, met the ASEX criteria for SD. These results, while specific to a particular tertiary clinic in a Western Canadian city, reiterate the importance of conducting assessments of sexual functioning with patients presenting with mental health concerns and for the training of clinicians in matters of assessing and treating SD^4,11^.

The findings that ADHD patients may suffer less from SD are intriguing. While the use of SSRIs for the treatment of depression have been found to contribute to SD, there is evidence that some ADHD treatments may prevent SD. The efficacy of psychostimulant medications for the management of ADHD has been well established^42^. Early insights into the association of stimulant use and SD revealed a potential for increased sexual functioning with acute stimulant dosing^43^. Bartlik and colleagues reviewed five cases of patients with ADHD and similarly found marked improvement in delayed or inhibited orgasm or ejaculation with low dosages of psychostimulants^44^. Future research should further explore the potentially protective effects of an ADHD diagnosis and/or associated psychostimulant treatments on SD.

This study has a number of limitations. Although the sample size is sufficient for classification tasks and machine learning-based pilot studies^34,35,45^, we were unable to identify statistically significant effects of a specific drug class, including SSRIs or SNRIs, predicting TESD as has been reported in the literature. In our sample, individual drug-related features reflecting the status of pre-existing treatments showed only a very weak correlation with SD (Table S2). However, we found removing all drug-related features from our feature pool led to a 4% decline in balanced accuracy. Thus, pre-existing drug treatments are important contributors for accurate SD prediction and inclusion of drug-related features in future large sample SD identification studies are warranted. Also, although we implemented nested cross-validation to reduce the risk of overfitting, overfitting risks may be inflated with a small sample size. A larger sample might help reduce overfitting and facilitate cross-referencing results with other statistical methods or machine-learning algorithms.

Furthermore, this is not a study on the general relationship between mental health disorders and sexual dysfunction. Therefore, the results cannot be generalized to all adult men and women with mental health disorders. The study was cross-sectional, allowing for correlational analysis but not causal attribution. The study sample was also non-randomized and we were unable to control for confounding variables such as disease severity, duration, comorbid disorders, medications and type of SD. However, the ML approach was optimized for predictive performance and while identified predictive risk factors should be interpreted as useful to guide clinical practice, other non-significant risk factors found in this study should not be summarily disregarded as null, pending future converging evidence with larger samples. Relatedly, the study cannot account for the effects of additional demographical, relational, and biological risk factors for sexual dysfunction that were not assessed. In addition, this study was conducted in a single clinic and the results may have limited generalizability. Finally, we did not assess for or obtain a definitive diagnosis of sexual dysfunction according to DSM criteria and instead relied on the ASEX, a validated but non-diagnostic tool; these results do, however, demonstrate the impetus of such assessments in conjunction with assessment for MDD. Future studies with large samples, multiple clinical sites, clinically validated SD diagnoses and clear drug utilization information will be necessary to address these limitations.

## Conclusion

This proof-of-concept pilot study found a high prevalence of SD in psychiatric outpatients, demonstrated the potential of ML-based applications to facilitate individual-level clinical screening of SD among mental health patients, and identified clinically relevant predictive risk factors. Taken together, these findings provide impetus for clinicians to be mindful of the high likelihood of SD in patients seeking mental health treatment, particularly women and those exhibiting depression.

## Supplementary Information

Supplementary Information.

## Data Availability

The datasets used and/or analyzed during the current study are available from the corresponding authors on reasonable request.
