# Supplementary material for: Individualized identification of sexual dysfunction of psychiatric patients with machine-learning
Source: Sci Rep. 2022 Jun 10;12:9599. doi: 10.1038/s41598-022-13642-y (PMC9187754; doi:10.1038/s41598-022-13642-y)
Supplement: Supplementary file 1 — Supplementary Information. [file 41598_2022_13642_MOESM1_ESM.docx]

**Supplementary Materials for *Individualized Identification of Sexual Dysfunction of Psychiatric Patients with Machine-learning*** (Liu, Hankey, Chokka, Chokka & Cao , 2022)

**Table S1. Summary of patient characteristics**

|  | With SD | | Non SD | | Between-group difference | |
| --- | --- | --- | --- | --- | --- | --- |
|  | Mean | SEM | Mean | SEM | t | Adjusted p |
| Age | 39.4 | 1.6 | 34.9 | 1.4 | 2.11 | 0.000 |
| Sex (Female) | 73.6% | 0.05 | 47.5% | 0.06 | 3.17 | 0.014 |
| MDD | 61.1% | 0.06 | 23.0% | 0.05 | 4.75 | 0.028 |
| BD | 12.5% | 0.04 | 19.7% | 0.05 | -1.13 | 0.130 |
| GAD | 51.4% | 0.06 | 44.3% | 0.06 | 0.82 | 0.389 |
| BPD | 27.8% | 0.05 | 21.3% | 0.05 | 0.86 | 0.420 |
| ADHD | 26.4% | 0.05 | 49.2% | 0.07 | -2.77 | 0.524 |
| No medication | 18.1% | 0.05 | 27.9% | 0.06 | -1.35 | 0.487 |
| SSRIs | 40.3% | 0.06 | 31.1% | 0.06 | 1.09 | 0.568 |
| Non-psychiatric medication | 34.7% | 0.06 | 23.0% | 0.05 | 1.49 | 0.550 |
| Other antidepressant | 29.2% | 0.05 | 23.0% | 0.05 | 0.81 | 0.529 |
| Stimulants | 9.7% | 0.04 | 9.8% | 0.04 | -0.02 | 0.491 |
| Benzodiazepines or hypnotics | 26.4% | 0.05 | 19.7% | 0.05 | 0.91 | 0.877 |
| Antipsychotics or anticonvulsants | 18.1% | 0.05 | 19.7% | 0.05 | -0.24 | 0.983 |

Descriptive summary of patient characteristics of the SD and non SD group. Independent sample t-tests were performed and presented with FDR (α =0.05) adjusted p values. SEM denotes standard error of the mean,

**Table S2 – Zero order correlations of features.**

|  |  | % of  N | 1 | 2 | 3 | 4 | 5 | 6 | 7 | 8 | 9 | 10 | 11 | 12 | 13 | 14 |
| --- | --- | --- | --- | --- | --- | --- | --- | --- | --- | --- | --- | --- | --- | --- | --- | --- |
| 1 | SD | 54.1% | 1 |  |  |  |  |  |  |  |  |  |  |  |  |  |
| 2 | Age | - | .18 | 1 |  |  |  |  |  |  |  |  |  |  |  |  |
| 3 | Sex (Female) | 61.7% | **.27** | -.15 | 1 |  |  |  |  |  |  |  |  |  |  |  |
| 4 | MDD | 43.6% | **.38** | **.25** | .13 | 1 |  |  |  |  |  |  |  |  |  |  |
| 5 | BD | 15.8% | -.10 | -.08 | .09 | -.30 | 1 |  |  |  |  |  |  |  |  |  |
| 6 | GAD | 48.1% | .07 | .02 | .11 | .00 | -.09 | 1 |  |  |  |  |  |  |  |  |
| 7 | BPD | 24.8% | .07 | .05 | .20 | .06 | .04 | -.10 | 1 |  |  |  |  |  |  |  |
| 8 | ADHD | 36.8% | -.24 | -.29 | -.10 | -.33 | .14 | -.27 | -.19 | 1 |  |  |  |  |  |  |
| 9 | No medication | 22.6% | -.12 | -.20 | .02 | -.18 | -.09 | -.09 | -.02 | .07 | 1 |  |  |  |  |  |
| 10 | SSRIs | 36.1% | .09 | .09 | .11 | **.22** | .10 | .15 | .00 | -.12 | -.41 | 1 |  |  |  |  |
| 11 | Non-psychiatric medication | 29.3% | .13 | **.24** | .07 | .10 | -.05 | .01 | .05 | -.05 | -.31 | .00 | 1 |  |  |  |
| 12 | Other antidepressants | 26.3% | .07 | **.30** | -.13 | **.23** | -.07 | -.06 | **.25** | -.21 | -.32 | -.09 | **.25** | 1 |  |  |
| 13 | Stimulants | 9.8% | .00 | -.12 | .00 | -.14 | .00 | -.16 | -.13 | **.27** | -.18 | -.04 | -.05 | -.08 | 1 |  |
| 14 | Benzodiazepines or hypnotics | 23.3% | .08 | .15 | .03 | -.05 | **.25** | .18 | .05 | -.09 | -.25 | .10 | .04 | .11 | -.12 | 1 |
| 15 | Antipsychotics or anticonvulsants | 18.8% | -.02 | .00 | -.10 | .00 | .16 | .11 | .12 | -.01 | -.26 | .00 | .03 | .15 | .04 | .14 |

Note. p <0.05 when r > 0.171. p < 0.05 when r > 0.223, p < 0.001 when r > 0.283, in bold font.
